# Part of the solution: A survey of community organisation perspectives on barriers and facilitating actions to Advance Care Planning in British Columbia, Canada

**DOI:** 10.1111/hex.13390

**Published:** 2021-12-14

**Authors:** Ellie G. Siden, Rachel Z. Carter, Doris Barwich, Eman Hassan

**Affiliations:** ^1^ Department of Medicine University of British Columbia Vancouver British Columbia Canada; ^2^ BC Centre for Palliative Care New Westminster British Columbia Canada; ^3^ Department of Medicine, Division of Palliative Care University of British Columbia Vancouver British Columbia Canada

**Keywords:** Advance Care Planning, awareness, British Columbia, community, engagement, nonprofits, public

## Abstract

**Background:**

Despite the established benefits of Advance Care Planning (ACP), engagement remains low in British Columbia. Since 2016, a growing number of community‐based nonprofits have offered ACP education. To date, no study has focused on the perspectives of nonprofits on ACP in British Columbia.

**Objective:**

This study aimed to identify barriers and facilitating actions to ACP as perceived by British Columbian nonprofits.

**Design:**

A mixed‐methods design was used. Data were collected through online surveys and telephone interviews.

**Setting and Participants:**

Staff and volunteers from British Columbian nonprofits that are providing or interested in providing public education on ACP were recruited for this study.

**Results:**

The lack of public awareness of ACP, the emotional difficulty of the conversation, the complicated ACP process, the belief that ACP is synonymous with completing a medical order form, the challenge of introducing ACP in different cultural contexts and the siloed approach to ACP education were rated as the most important barriers to ACP engagement. The most important facilitating actions were developing clear messages, improving ACP literacy, reframing ACP as part of life planning, simplifying ACP documentation and transfer, integrating ACP conversations into clinical practice and better collaboration between the health system and nonprofits.

**Discussion:**

This study identifies numerous opportunities to improve ACP engagement in British Columbia from a community lens. To maximize ACP engagement, community‐led ACP education should be offered in coordination with the health system.

**Conclusion:**

Community‐led ACP education as well as collaboration and consultation with nonprofits are part of the solution to the low ACP engagement in British Columbia.

**Public Contribution:**

Study participants, including staff and volunteers at nonprofits, are members of the public.

## 1 BACKGROUND

Advance Care Planning (ACP) is a process that helps adults to reflect on and share their personal values, goals and preferences as they relate to their future healthcare.^1^ Ideally, ACP should begin before health crises happen, so that it can inform ‘goals of care’ conversations and healthcare decision‐making throughout the person's journey with illness. The goal of ACP is to help people be prepared to make informed healthcare choices and have their healthcare wishes known and respected. There is a growing body of evidence that ACP is associated with increased patient and family satisfaction, decreased patient anxiety, reduced stress on families and reduced unwanted aggressive treatments and hospital deaths.^2^, ^3^

Despite the established benefits of ACP, a 2019 survey conducted by the Canadian Hospice and Palliative Care Association shows that ACP engagement is relatively low among Canadians. While 80% of survey participants think that it is important to do ACP, only about a third have discussed it with their family, and less than one in ten have talked with a healthcare provider about their wishes. Additionally, less than 20% of the survey respondents had documented their healthcare wishes.^4^ Low rates of ACP engagement are unfortunately not unique to Canada.^5^ A recent Australian survey found that only 15% of adults had completed ACP documentation,^6^ while studies in Northern Ireland and Singapore found rates of having had an ACP discussion to be 7% and 12.5%, respectively.^7^, ^8^

ACP education and conversations have generally been restricted to the health system, but in a recent study, healthcare providers reported insufficient time and their own lack of knowledge as significant barriers.^9^ Over the last decade, there has been an increasing recognition of the importance of community engagement in ACP promotion and education.^10^, ^11^, ^12^, ^13^ Recent articles highlight the necessity of viewing ACP through a public health lens, and the potential for community‐based nonprofits to normalize ACP conversations in the community and integrate it in a more timely manner into peoples' lives.^11^, ^12^, ^13^

Throughout this paper, we will define ‘non‐profits’ as community‐based nonprofits serving or interested in supporting people affected by serious illness, death or bereavement. Examples of nonprofits in Canada are hospice societies, seniors' centres, ethnocultural societies, faith‐based organisations and disease support organisations. These organisations are well connected with the people they serve and well situated in their communities to provide low‐cost, widely accessible information and resources about ACP. In addition to delivering ACP education sessions, many of these nonprofits promote ACP through their websites, distribute resources to local clinics and care facilities or host death‐positive conversations. The resources and programmes that they offer are well received, as they are tailored to the needs of their local populations and driven by trained volunteers at no or very low cost. Volunteers are integral to the structure and function of many ACP initiatives worldwide.^14^

In the spring of 2016, the British Columbia Centre for Palliative Care introduced Canada's first community‐led ACP education programme. Through this programme, the British Columbia Centre for Palliative Care has trained and equipped over 200 community‐based nonprofits across British Columbia with toolkits, seed funding and coaching to help them deliver group conversation games and information sessions about ACP to the public. The sessions were designed to raise public awareness of the importance of ACP for every capable adult, prepare and support individuals to initiate ACP conversations with their family and healthcare providers and provide information about the available options in British Columbia to legally document personal healthcare wishes.

This study is part of the British Columbia Centre for Palliative Care's continuous efforts to understand current barriers to ACP engagement and the most important actions needed to facilitate ACP uptake in British Columbia, in this case from the perspective of nonprofit organisations. To date, no study has focused on the perspectives of community nonprofits on low ACP uptake in British Columbia. The findings will inform current and future provincial ACP policies and efforts towards what can best support the public uptake of ACP in partnership with the British Columbian nonprofit sector.

## 2 METHODS

A mixed‐methods approach was used to examine the perspectives of British Columbian nonprofits on public engagement in ACP. Quantitative and qualitative data were collected from staff and volunteers of British Columbian nonprofits who were asked to prioritize a list of facilitating actions recommended by the 2019 Pan‐Canadian ACP Framework,^15^ and a list of barriers identified from a literature search.

This study considered three research questions from the perspective of British Columbia nonprofits: (1) What are the most important barriers to ACP engagement in British Columbia? (2) What are the most important actions to facilitate ACP uptake in British Columbia? and (3) Has the COVID‐19 pandemic changed the ACP needs and activities, and if so, how can nonprofits be helped to support their communities with these needs? The participants' responses to these questions were collected through an online survey, followed by one‐on‐one telephone interviews with those who agreed to be interviewed. The surveys and interviews were conducted during June and July 2020. For this paper, we report only on the first two questions, as we believe that the question regarding the COVID‐19 pandemic is less likely to be applicable going forward.

Ethics approval was obtained from the University of British Columbia Behavioral Research Ethics Board (H16‐00044). Consent was obtained for both the online survey and qualitative interviews.

### 2.1 Participants

We emailed the invitation and web link to the online survey to 355 contacts of staff or volunteers affiliated with nonprofits based in British Columbia. These nonprofits were identified through two sources: (1) the British Columbia Centre for Palliative Care's community network list. Many of the nonprofits on this list actively engage their communities in ACP. (2) A list populated through online searches of British Columbian nonprofits, a part of whose mandates or services include public education on health topics. This list included seniors' centres, community health centres, libraries and faith‐based organisations, among others. To maximize the survey reach, we encouraged the contacts on the two lists to forward the survey invitation email and web link to similar organisations.

In the invitation, we asked only those organisations actively involved in ACP promotion or education or those with knowledge of ACP issues in their communities to complete the survey. Follow‐up telephone interviews were offered to everyone who had completed the survey. Demographic information specific to the nonprofits, but not to the individuals, was collected as we asked each participant to speak from the perspective of their organisation.

### 2.2 Survey design

We based the survey structure on previously published studies and national frameworks that addressed barriers to and enablers of ACP. To contextualize the survey items to British Columbian nonprofits and assure content validity and wording clarity, we consulted a palliative care physician (D. B.), ACP researchers (D. B., R. C., E. H.) and community engagement and development experts at British Columbia Centre for Palliative Care (K. K., M. J.).

The survey started with an introductory component that collects demographic information about the participating nonprofits, followed by two questions about the organisations' perceived barriers and facilitating actions to ACP engagement. The question about barriers to ACP included a list of 15 items representing the most frequently mentioned barriers in recent Canadian literature from a patient/caregiver perspective.^10^, ^16^, ^17^, ^18^, ^19^, ^20^, ^21^, ^22^, ^23^, ^24^ For details about this literature search, please see Appendix SA. We restricted our search to Canadian literature to maximize its applicability to our population. For this question, participants were asked to review the list, select a total of six of the most common barriers that they have observed in their communities and rank the selected items from one (the most) to six (the least) important.

The question on ACP facilitating actions included a list of provincial‐focused actions recommended by the 2019 Pan‐Canadian ACP Framework.^12^ We examined each action for clarity, concision and nonredundancy; each item had to be understandable on its own and clearly different from the other actions. This process resulted in some rewordings and consolidations and a final list of 33 items. From this list, participants were asked to select and rank a total of eight of the most important actions needed to increase public engagement in ACP in their communities. In the barriers and facilitating actions questions, participants were given the opportunity to write additional high‐priority barriers and actions not included in the lists. The last four questions in the survey were designed to collect information about the impact of COVID‐19 on ACP needs and activities.

The version of the online survey that was sent to nonprofits whose involvement in ACP activities was unknown to us included several additional screening questions at the beginning to ensure that the survey‐taker was familiar with ACP needs and issues in their communities. See Appendix SB for both versions of the online survey.

### 2.3 Interviews

One‐on‐one telephone interviews were conducted by E. S., a female medical student, with those who indicated in the survey their interest in being interviewed. The purpose of the interviews was to give participants the opportunity to elaborate on their survey responses and articulate barriers and facilitating actions to ACP in their own words. Before beginning the interview, E. S. introduced herself and reviewed the study's objectives with the interviewee. All interviews were recorded with permission, transcribed verbatim and then anonymized. No repeat interviews were undertaken. Field notes were not taken during the interviews, and transcripts were not returned to interviewees for comment. Interviews lasted 14–32 min. See Appendix SC for the interview script.

### 2.4 Analysis

Overall rank order for the barriers and facilitating actions questions was determined using a weighted calculation. For example, participants were asked to rank six barriers, so the most important barrier was assigned six points. If one participant ranked a barrier as most important (six points) and another participant ranked the same barrier as the second most important (five points), then it would be assigned a cumulative 11 points. The same system was used for the facilitating actions, with a maximum of eight points. Nonranked items were assigned a zero.

Free‐text survey questions and interview transcriptions were analysed using NVivo12. All materials were doubly coded, first deductively using the question options as themes and then again using interpretive description and an inductive process.^16^ This enabled us to develop a narrative explanation of participants' rankings of barriers and facilitating actions as well as to elucidate any themes outside of these existing categories. Initial coding was performed by E. S., and reviewed by R. C. Following coding, E. S. and R. C. met to discuss and reach consensus on emerging themes. Saturation was deemed to have been reached once no new major themes were being discovered upon coding.^25^

## 3 RESULTS

### 3.1 Participant characteristics

Fifty‐seven nonprofits completed the online survey, yielding a maximal survey response rate of 16%, although the exact rate cannot be calculated due to snowball sampling. Seventeen (30%) self‐selecting survey participants were interviewed. Surveys and interviews were conducted in English only. The characteristics of the participating organisations are summarized in Table 1. Data were not collected as to why some potential participants did not respond.

**Table 1:** Organisation characteristics of survey participants

	Survey participants (*n* = 57), *N* (%)	Interviewees (*n* = 17), *N* (%)
Organisation type		
Hospice society	25 (44)	9 (53)
Organisation supporting seniors in the community	19 (33)	2 (12)
Organisation that supports people affected by a specific disease or illness	5 (9)	3 (18)
Culturally specific services	1 (2)	1 (6)
Other^a^	7 (12)	2 (12)
Organisation location		
Metropolitan areas (Lower Mainland)	8 (14)	3 (18)
Rural (Fraser Valley, Vancouver Island, Interior Region)	35 (61)	10 (59)
Remote (Sunshine Coast, Northern BC)	5 (12)	2 (12)
Provincial organisations	7 (12)	2 (12)

^a^ Community health centres, community death‐caring network, organisation supporting healthy lifestyles and healthcare in the community; faith‐based organisation; community‐based programming through nature‐based and community‐based learning; and settlement and immigration.

### 3.2 Most important barriers to ACP

The rank order for the most important barriers to ACP engagement in British Columbia from the perspectives of nonprofits is shown in Table 2. The top six barriers identified through the qualitative analysis are listed below. While there is considerable overlap with those found in quantitative analysis, several new themes are also introduced.

1. **Lack of public awareness** (also top‐ranked in the quantitative analysis)
  The public's lack of awareness is the most important barrier to engagement in ACP according to the participants. One participant described that, before she began ACP education, ‘people hadn't heard of it. They didn't know what it was’ This was felt to be particularly true for new immigrants, whose home countries may have had different processes and cultures surrounding healthcare decision‐making.
2. **Emotional barriers** (also top‐ranked in the quantitative analysis)
  The public's discomfort with discussing end‐of‐life matters was described as a major barrier to ACP. Many described a culture of ‘denial, simply not wanting to go there’. When people in this death‐denying culture were forced to confront their mortality, they often did so with fear. Occasionally, this fear provided a catalyst for ACP discussions:
*I think that [COVID] has kind of heightened people's awareness. It's kind of like…this has been on the side burner for the last couple of months or the last couple of years. We really need to get on this because we don't really know what could happen*.
  However, the public's discomfort with the topic of end‐of‐life was occasionally such that it prevented them from benefiting from ACP education:
*People will come back [after they have attended education] and say, ‘Well, nobody said anything’… They're just not available to hear it*.
  Healthcare providers were believed ‘to be like everyone else. They don't really want to talk about [death], either’. In contrast, this reluctance to discuss death was not reported as an issue with staff and volunteers at nonprofits.
3. *Confusing terminology and complicated process* (also top‐ranked in the quantitative analysis)
  The terminology used in ACP information materials and guidebooks was often confusing to members of the public and contributed to a lack of understanding. Participants noted that ‘a lot of people get quite flustered by the terminology’*,* which seemed to change frequently, involved legal jargon and was inconsistent between provinces. The ACP process was also thought to be too complicated and involve many steps. This made it challenging to complete for members of the public.
  Many participants also indicated that the length and complexity of the current provincial ACP guide were ‘overwhelming’, and a barrier to the public's engagement with ACP. However, because of the legal nature of the documents involved with ACP, participants still felt obligated to use it.
4. *Belief that ACP is a one‐time conversation to specify medical orders* (also top‐ranked in the quantitative analysis)
  Healthcare providers, especially physicians, were described as conflating ACP with Medical Order for Scope of Treatment (MOST) or Do‐Not‐Resuscitate (DNR) forms, or disregarding ACP in favour of these forms.
*Here, they won't even put a flag on my computerized records saying that I have an Advance Care Plan…*
*Well, we can't do that because we do the MOST thing*.
*Well, okay, but they're really different!*
  This conflation has resulted in healthcare providers leading fewer true ACP discussions, and in some cases, even the refusal of patients' direct requests to discuss ACP.
  Participants also reported similar confusion amongst the public around DNR forms, wills and ACP. This impeded ACP because some people thought that they had already completed it, despite that not being true.
*There is a really big feeling amongst a great number of people in the community that once they've made a Will, they're done. Or maybe if they do sign a DNR at a long‐term‐care facility. But they don't really understand what ACP is all about and what kinds of things you might think about*.
5. *Challenge related to different cultural contexts* (emerged in the qualitative analysis only)
  The experience was that ACP education and materials needed to be adjusted for culturally diverse populations. Culture is known to influence ACP receptiveness as well as people's views of illness, quality of life, decision‐making and treatment preferences.^26^ The lack of culturally adapted resources often presented a challenge for ACP educators and healthcare providers. This resulted in the delivery of ACP education that was subpar or ineffectual.
*A lot of places, it's a cultural decision, it's a family decision, how you move forwards in terms of treatment. And we don't necessarily recognize that*.
6. *Siloed approach to ACP education* (emerged in the qualitative analysis only)

**Table 2:** Weighted ranking of most important barriers to Advance Care Planning in British Columbia from a nonprofit perspective

Barrier	Weighted score	Number of participants (*n* = 57), *n* (%)
Complete lack of awareness of ACP on the part of the individual	159	31 (55)
Emotional difficulty of the conversation	155	38 (67)
Confusion or lack of knowledge about how to begin or perform ACP on the part of the individual	152	37 (65)
The belief that ACP is a one‐time conversation to specify a DNR designation	130	31 (55)
Belief that ACP is redundant because the family already knows one's wishes	105	34 (60)
Conflict with family members or hesitancy of family members	89	28 (49)
People do not understand the progression or the seriousness of their own illness	70	19 (33)
The complex terminology involved with ACP is hard to understand	60	18 (32)
Healthcare provider lack of time	56	25 (44)
Belief that planning for or discussing death brings bad luck or is taboo	36	12 (21)
Healthcare provider lack of tact or conversation skill	33	12 (21)
Healthcare provider lack of knowledge	29	13 (23)
Mistrust of the medical system	28	11 (19)
Lack of family with whom to discuss wishes	27	16 (28)
People do not speak English and do not have access to ACP resources in their own language	19	6 (11)

Abbreviations: ACP, Advance Care Planning; DNR, Do‐Not‐Resuscitate.

Nonprofits saw their ACP education efforts as siloed from the healthcare system. Many did not know how exactly ACP was being conducted within the health system, and some expressed anxiety that they were duplicating efforts or distributing contradictory information. Additionally, some participants reported that health authorities and healthcare providers had little knowledge about or interest in their services.

Participants wished for better collaboration to deliver efficient, coordinated ACP education to the public. Differences in structure between the health authorities and the community organisations made this collaboration occasionally challenging.

*From a community perspective, we have more flexibility and can do things faster… If it's not a priority of theirs until year two and you're ready to go year one, does that mean you don't do anything?*

### 3.3 Most important facilitating actions

The list of actions ranked by study participants as high‐priority facilitating actions to ACP are shown in Table 3, and a complete list is provided in Appendix SD. The top six facilitating actions identified through the qualitative analyses are below. Similar to the barriers, the qualitative analysis of facilitating actions largely reflected the quantitative survey findings while also introducing several new ideas.

1. Develop clear, simple messages (also top‐ranked in the quantitative analysis)
  Participants wanted a ‘simple, central’ ACP tool that is short, clear and comprehensive. A clear guide would address confusion related to the ACP process and terminology and improve uptake rates.
  Many nonprofits wished that the current provincial ACP guide included clearer and simpler messages and easier steps. Given its official nature, the provincial guide was seen as the ACP resource that they ‘should’ be using, despite its non‐user‐friendly nature.
2. *Improve ACP literacy* (also top‐ranked in the quantitative analysis)
  Through the qualitative analysis, participants reported that nonprofits are well equipped to deliver high‐quality ACP education to the public and are eager to continue sharing their knowledge with their communities.
*We have some very elderly, frail people come to our courses. And the difference in them when they left was just extraordinary. It was like they'd been holding these thoughts and worries and upsets. And it made them realize…they could actually do this according to what they wanted and not be forced by their family*.
  Community organisations were thought to be able to provide more accessible, less ‘authoritative’ ACP education than that delivered through the healthcare system.
*There's a perception that it needs to be led by some form of healthcare professional, and I don't think that is the solution. I think that there could be more collaborative efforts in bringing it down to a level of comfortablity [sic] for people to start talking and being not afraid to talk about it*.
  Several participants provided examples of the ways in which they had used their knowledge of their community to provide targeted ACP education. This has resulted in ACP education that had greater relevance to and impact on their specific populations.
*I'll ask an elder to share a story of their experience in the hospital. And I do some pre‐work with them first, so they know to talk about what they wish they would have known… And I think when [the community hears] about [ACP] coming from an elder about how things could have been done differently, that's how I introduce the topic of ACP… I find it's different in every community I work in*.
3. *Reframe ACP as part of life planning* (also top‐ranked in the quantitative analysis)
  Most participants strongly associated ACP conversations with discussions of end‐of‐life and death. Many therefore thought that the emotional hurdle of ACP was based on difficulties discussing death, and proposed that ‘if [death and end‐of‐life] were part of our daily conversation, they [become] just normal, they're not scary anymore and we can talk about it, and talk over the things that we're worried about’.
  Many participants expressed that ACP conversations should also be begun earlier, ‘It needs to be okay to talk to our young children about death and dying, because we are all leaving one day’.
  Including for‐profit and not‐for‐profit community organisations in the ACP process was thought to be another way of introducing ACP earlier and enabling the integration of ACP into peoples' lives.
*You could work it into education practices, in terms of parent teacher associations. You would put out information when your child registers for school, say in kindergarten. And you'd put out a package and include in that package information about ACP*.
4. *Simplify the documentation and transfer of ACP conversations across care settings* (also top‐ranked in the quantitative analysis)
  The participants expressed the importance of streamlining the ACP documentation process so that the person's healthcare wishes and instructions are accessible when needed. This means documenting ACP conversations electronically, training healthcare providers to document ACP and ensuring that this documentation is available at all hospitals and clinics in British Columbia. Simplified documentation and transfer would decrease confusion around the ACP process and terminologies for both the public and healthcare providers.
*It should be all electronic right now. And the Advance Care Plan stuff isn't yet in the electronic filing system*.
5. *Integrate ACP conversations into the scope of practice for all healthcare providers* (also top‐ranked in the quantitative analysis)
  Approaching ACP as a conversation, instead of as a form or a checkbox—‘[letting] people start wherever they're going to start thinking about their end’—, was also seen as a beneficial and potentially more accessible means of conducting ACP. Doing so can allow not only the individual but also their family to contemplate the meaning and importance of their life and their values. Additionally, framing ACP as a conversation was thought to make ACP more accessible to some healthcare professionals, as well as to people who are mistrustful of institutional settings. ACP conversations initiated by family physicians were ‘[viewed] with a different lens [than those with non‐profits] because it's such a trusting, authoritative place for people, within their care’. Physicians' support for and initiation of the ACP conversation were felt to be crucial, especially for some minority groups, who may be more inclined to follow a doctor's recommendations than that of a community organisation.
6. *Better collaboration between the health system and nonprofits* (emerged in the qualitative analysis only)

**Table 3:** Weighted ranking of most important actions to increase Advance Care Planning in British Columbia from a nonprofit perspective

Facilitator	Weighted score	Number of participants ranking facilitator (*n* = 48), *n* (%)
Develop clear, simple messages with and for target audiences	151	21 (44)
Improve ACP literacy (e.g., walk people through ACP steps, increase training for providers)	150	23 (48)
Reframe ACP as part of life planning (e.g., build opportunities for ACP into life milestones)	140	22 (45)
Simplify the documenting and transferring of ACP conversations	108	21 (44)
Define core ACP competencies and integrate them into the scope of practice, and both initial and ongoing training for all healthcare providers	88	17 (35)
Work together with local partners to develop and adapt relevant tools and resources	84	19 (40)
Establish standards for having ACP conversations, documenting and accessing them and translating them into medical orders	81	20 (42)
Develop a network of key partners that already help people consider their values and think about the future (e.g., lawyers, faith‐based organisations, financial planning services)	70.5	20 (42)
Provide cultural safety and humility training to healthcare providers as a way to support ACP with culturally diverse communities and populations	68.5	15 (31)
Identify champions who can be mobilized to promote ACP awareness and education (e.g., mentorship programmes)	66	15 (31)

Abbreviations: ACP, Advance Care Planning; DNR, Do‐Not‐Resuscitate.

Participants also thought that better collaboration with the health system could help them distribute their resources to a wider public. Several suggested collaborations through which ‘local family health teams [could] collaborate with…local community organizations, where [the] community organization is doing [an] ACP planning workshop once a week, and [the] physician is referring their patient to this workshop’. Ideas such as these were felt to potentially save healthcare providers' time while ensuring good‐quality ACP for their patients.

## 4 DISCUSSION

This study is among the first to rank barriers and facilitating actions to public engagement in ACP from the perspectives of nonprofits. A few studies, including one British Columbian study, have included nonprofits among others in their explorations of stakeholders' perspectives on end‐of‐life and ACP, but none rank the importance of the identified barriers or enablers.^10^, ^12^, ^27^, ^28^ Others have ranked barriers and facilitating actions to ACP, but from the perspectives of healthcare providers or the public only.^9^

The barriers and facilitating actions that nonprofits identified in our study as ‘most important’ were also found to be important in studies focusing on the perspectives of the public.^12^, ^17^, ^18^, ^21^, ^22^ As we developed the options in the survey from the literature, it makes sense that our findings supported the existing literature. A 2019 survey of the Canadian public, with provincial breakdowns, resulted in similar ‘top’ barriers and facilitating actions for British Columbia; ‘I do not feel it is relevant to me’ and ‘I do not know where to go for information or advice’ were selected as the items making it difficult to complete ACP, whereas ‘more personal time to reflect on my wishes’ and ‘more resources to provide guidance on ACP’ were chosen as the items that would make ACP easier. The only true outlier seems to be the wish for ‘more personal time to reflect on my wishes’, which may have implications for how much time to schedule between public information activities with more than one session.

In our study, most participants closely associated ACP with end‐of‐life and believed that the discomfort around these conversations was at heart a fear of considering mortality. This correlation of ACP with end‐of‐life is slightly at odds with recent consensus definitions of ACP, which describe ACP as a process of communicating’ personal values, life goals and preferences’ for future medical care, including serious and chronic illness.^1^, ^29^

‘Reframing ACP as part of life planning,’ including the engagement of community organisations, has been proposed by several studies as a potential solution to these initial feelings of discomfort, wherever they may originate.^10^, ^11^, ^12^, ^30^ Our study confirms that many nonprofits find their role in ACP promotion and education to be a promising solution to the associated emotional barriers, and in particular, to the fear of death. Many organisations are already engaged in the ‘normalization’ of discussions around issues associated with serious illness and end‐of‐life care, and believe that their use of personal stories and knowledge of their communities make them uniquely suited to support ACP engagement and help people have their healthcare wishes known and respected.

A new barrier that emerged in the qualitative analysis is the siloed nature of the ACP education efforts of nonprofits and the health system. In their study of services provided by British Columbian hospice societies, Gyapay et al.^31^ noted similar challenges in keeping healthcare providers informed of hospice services. It is possible that previous studies that combined public perceptions with those of community organisations may have obscured these viewpoints.^10^, ^12^

Collaborations between nonprofits, healthcare systems and academic institutions have been shown to have fairly consistent positive effects.^32^ Several international studies suggest positive impacts of these collaborations on public ACP education.^27^, ^33^ Healthcare provider willingness to learn about and from nonprofits providing ACP education is vital to future collaborations between the health system and nonprofits. Study participants eagerly proposed ideas such as a referral system whereby healthcare professionals might refer patients to a community‐based ACP workshop. Even having a central hub for health system updates on ACP practices and procedures might allow nonprofits and healthcare professionals to coordinate efforts more easily. Ideas such as these are not specific to the health system, and could readily be enacted in a variety of locales to better support nonprofits and improve community ACP engagement.

An additional barrier that emerged during the qualitative analysis was the challenge of introducing ACP across different cultural contexts. This is a well‐established barrier in Canada as well as internationally, and many cultural factors are known to influence ACP receptiveness.^34^ However, the steps taken to mitigate this barrier vary by location, depending on the common cultural groups in the community. The British Columbia Centre for Palliative Care has recently codeveloped ACP resources adapted and translated for the South Asian and Chinese communities, in collaboration with members of those communities. After the successful adaptation of the Serious Illness Conversation Guide for Indigenous communities, the British Columbia Centre for Palliative Care is in the process of adapting it to culturally diverse populations.^35^ Resources such as these, as well as the involvement of diverse nonprofits employing and serving various cultural groups and increased cultural‐sensitivity training for healthcare providers, may begin to address this important barrier.

Finally, the inclusion and welcoming of a wide variety of nonprofits into ACP education work may have myriad benefits including, as mentioned by our study participants as well as other literature, encouraging better targeting of ACP education to marginalized populations,^27^ normalizing ACP and therefore lessening the emotional barriers^11^ and sparing the healthcare system time and money. While some participants expressed eagerness to include other nonmedical providers or for‐profit organisations, such as lawyers, in the ACP process, others emphasized the unique accessibility provided by nonprofits. However, all these benefits can only be fully realized if other steps also occur, such as an improved provincial ACP guide, better healthcare provider education differentiating medical orders forms from ACP and improved ACP documentation.

The mixed‐methods study design allowed us to survey and interview participants from all areas of British Columbia. However, our study does have some limitations. First, as in many qualitative studies, the small sample size and geographic context of our study, including a literature search limited to Canadian literature, may limit the generalisability of our findings. Second, responses to both qualitative and quantitative findings may have been biased by the options that we offered on the survey. Third, we asked organisations to volunteer to fill out our survey and take part in our interviews. It is therefore likely that our sample is biased towards those organisations most invested in ACP, especially given our survey's maximum response rate of 16%. Our data may not therefore represent the full spectrum of barriers or facilitating actions to ACP or the priorities of nonprofits less experienced in ACP. Fourth, nonprofits supporting ACP in British Columbia are a heterogeneous group, and what is a barrier for one may not apply for another. Our study included a larger number of hospice societies than other organisation types, which may bias our conclusions towards the perspectives of hospice societies. However, the fact that we excluded for‐profits in our survey makes our study less heterogeneous than others.^10^, ^11^, ^12^ Finally, we conducted our study during the COVID‐19 pandemic, which may have influenced responses.

## 5 CONCLUSION

This study identifies numerous opportunities to increase and improve ACP according to non‐profits engaging in ACP education in British Columbia. It offers new insights into ACP from both a British Columbian and a community lens, and encourages, among other things, greater collaboration with and inclusion of community organisations in the ACP landscape in British Columbia. Findings from this study should be used to inform existing and future ACP policies across the province, and may be used to provide guidance on working with community groups and ACP on a national and international level. Research into ACP barriers and facilitating actions has largely limited itself to the clinician–patient dichotomy. Further inquiry into the needs of nonprofits, as the ‘middle men’ in ACP education, will be necessary to fully incorporate them into ACP processes. The inclusion of these ‘new’ voices can continue to offer novel and effective ways to increase and improve ACP in British Columbia and beyond.

## CONFLICT OF INTERESTS

The authors declare that there are no conflict of interests.

## Supporting information

Supporting information.

## Data Availability

The data that support the findings of this study are available from the corresponding author upon reasonable request.

## References

[1] Sudore RL , Lum HD , You JJ , et al. Defining advance care planning for adults: a consensus definition from a multidisciplinary Delphi panel. J Pain Symptom Manage. 2017;53(5):821‐832.e1. 10.1016/j.jpainsymman.2016.12.331 28062339 PMC5728651

[2] Van Wert R , Wallace E . Impact of advance care planning interventions on patient and family satisfaction: a systematic review and descriptive analysis (S777). J Pain Symptom Manage. 2018;55(2):698‐699. 10.1016/j.jpainsymman.2017.12.431

[3] Bischoff KE , Sudore R , Miao Y , Boscardin WJ , Smith AK . Advance care planning and the quality of end‐of‐life care in older adults. J Am Geriatr Soc. 2013;61(2):209‐214. 10.1111/jgs.12105 23350921 PMC3760679

[4] CHPCA, Nanos. *Canadians are thinking about end‐of‐life care but few have taken action*—CHPCA Survey Summary; 2019.

[5] Jeong S , Barrett T , Ohr SO , Cleasby P , Davey R , David M . Prevalence of advance care planning practices among people with chronic diseases in hospital and community settings: a retrospective medical record audit. BMC Health Serv Res. 2021;21(1):303. 10.1186/s12913-021-06265-y 33820535 PMC8022421

[6] White BP , Willmott L , Tilse C , et al. Prevalence of advance care directives in the community: a telephone survey of three Australian States. Intern Med J. 2019;49(10):1261‐1267. 10.1111/imj.14261 30785233

[7] McIlfatrick S , Slater P , Bamidele O , Muldrew D , Beck E , Hasson F . ‘It's almost superstition: If I don't think about it, it won't happen’. Public knowledge and attitudes towards advance care planning: A sequential mixed methods study. Palliat Med. 2021;35(7):1356‐1365. 10.1177/02692163211015838 34000901 PMC8267083

[8] Ng QX , Kuah TZ , Loo GJ , et al. Awareness and attitudes of community‐dwelling individuals in Singapore towards participating in advance care planning. Ann Acad Med Singapore. 2017;46(3):84‐90. http://www.ncbi.nlm.nih.gov/pubmed/28417132 28417132

[9] Howard M , Bernard C , Klein D , et al. Barriers to and enablers of advance care planning with patients in primary care: survey of health care providers. Can Fam Physician. 2018;64(4):e190‐e198. 29650621 PMC5897087

[10] Banner D , Freeman S , Kandola DK , et al. Community perspectives of end‐of‐life preparedness. Death Stud. 2019;43(4):211‐223. 10.1080/07481187.2018.1446060 29498611

[11] Prince‐Paul M , DiFranco E . Upstreaming and normalizing advance care planning conversations—a public health approach. Behav Sci. 2017;7(2):18. 10.3390/bs7020018 PMC5485448 28417931

[12] Biondo PD , King S , Minhas B , Fassbender K , Simon JE . How to increase public participation in advance care planning: findings from a World Café to elicit community group perspectives. BMC Public Health. 2019;19(1):1‐12. 10.1186/s12889-019-7034-4 31159829 PMC6547442

[13] Waller A , Sanson‐Fisher R , Ries N , Bryant J . Increasing advance personal planning: the need for action at the community level. BMC Public Health. 2018;18(1):606. 10.1186/s12889-018-5523-5 29739369 PMC5941331

[14] Sellars M , Simpson J , Kelly H , et al. Volunteer involvement in advance care planning: a scoping review. J Pain Symptom Manage. 2019;57(6):1166‐1175.e1. 10.1016/j.jpainsymman.2019.02.031 30853554

[15] Canadian Hospice Palliative Care Association. Advance care planning in Canada: A Pan‐Canadian Framework; 2019. https://www.advancecareplanning.ca/resource/advance-care-planning-framework/. Accessed September 1, 2021.

[16] Phillips CR , MacNab CJ , Loewen LM . Advance care planning and chronic kidney disease: what do patients know and what do they want? Nephrol Nurs J, 45(6):513‐523. http://www.ncbi.nlm.nih.gov/pubmed/30585708 30585708

[17] Peck V , Valiani S , Tanuseputro P , et al. Advance care planning after hospital discharge: qualitative analysis of facilitators and barriers from patient interviews. BMC Palliat Care. 2018;17(1):127. 10.1186/s12904-018-0379-0 30518345 PMC6282276

[18] Shaw M , Hewson J , Hogan DB , Raffin Bouchal S , Simon J . Characterizing Readiness for advance care planning from the perspective of residents, families, and clinicians: an interpretive descriptive study in supportive living. Gerontologist. 2018;58(4):739‐748. 10.1093/geront/gnx006 28329800

[19] Taneja R , Faden LY , Schulz V , et al. Advance care planning in community dwellers: a constructivist grounded theory study of values, preferences and conflicts. Palliat Med. 2019;33(1):66‐73. 10.1177/0269216318803487 30284950

[20] Lum HD , Jordan SR , Brungardt A , et al. Framing advance care planning in Parkinson disease. Neurology. 2019;92(22):e2571‐e2579. 10.1212/WNL.0000000000007552 31028124 PMC6556088

[21] Carbonneau M , Davyduke T , Spiers J , Brisebois A , Ismond K , Tandon P . Patient views on advance care planning in cirrhosis: a qualitative analysis. Can J Gastroenterol Hepatol. 2018;2018:1‐8. 10.1155/2018/4040518 PMC6069582 30079330

[22] Hutchison LA , Raffin‐Bouchal DS , Syme CA , Biondo PD , Simon JE . Readiness to participate in advance care planning: a qualitative study of renal failure patients, families and healthcare providers. Chronic Illn. 2017;13(3):171‐187. 10.1177/1742395316675023 28133991

[23] de Vries B , Gutman G , Humble Á , et al. End‐of‐life preparations among LGBT older Canadian adults: the missing conversations. Int J Aging Hum Dev. 2019;88(4):358‐379. 10.1177/0091415019836738 30871331

[24] Abdul‐Razzak A , Sherifali D , You J , Simon J , Brazil K . ‘Talk to me’: a mixed methods study on preferred physician behaviours during end‐of‐life communication from the patient perspective. Health Expect. 2016;19(4):883‐896. 10.1111/hex.12384 26176292 PMC5152726

[25] Fusch P , Ness L . Are we there yet? Data saturation in qualitative research. Qual Rep. 2015;20(9):1408‐1416. 10.46743/2160-3715/2015.2281

[26] Con A . *Cross‐cultural considerations in promoting advance care planning in Canada*; 2008. http://www.bccancer.bc.ca/NR/rdonlyres/E6F649B9-761C-4C51-89E0-C2F0834B8DCC/43096/AConAdvanceCarePlanning.pdf. Accessed September 1, 2021.

[27] Phipps EJ , True G , Murray GF . Community perspectives on advance care planning: report from the Community Ethics Program. J Cult Divers. 2003;10(4):118‐123. 15000054

[28] LeBaron VT , Smith PT , Quiñones R , et al. How community clergy provide spiritual care: toward a conceptual framework for clergy end‐of‐life education. J Pain Symptom Manage. 2016;51(4):673‐681. 10.1016/j.jpainsymman.2015.11.016 26706624 PMC5987222

[29] Rietjens JAC , Sudore RL , Connolly M , et al. Definition and recommendations for advance care planning: an international consensus supported by the European Association for Palliative Care. Lancet Oncol. 2017;18(9):e543‐e551. 10.1016/S1470-2045(17)30582-X 28884703

[30] Fried TR , Bullock K , Iannone L , O'Leary JR , Lannone L , O'Leary JR . Understanding advance care planning as a process of health behavior change. J Am Geriatr Soc. 2009;57(9):1547‐1555. 10.1111/j.1532-5415.2009.02396.x 19682120 PMC2783892

[31] Gyapay J , Freeman S , Flood D . An environmental scan of caregiver support resources provided by hospice organizations. J Palliat Care. 2020;35(3):135‐142. 10.1177/0825859719883841 31838934

[32] Anderson LM , Adeney KL , Shinn C , Safranek S , Buckner‐Brown J , Krause LK . Community coalition‐driven interventions to reduce health disparities among racial and ethnic minority populations. Cochrane Database Syst Rev. 2015(6):9–28. 10.1002/14651858.CD009905.pub2 PMC10656573 26075988

[33] Cortez DM , Harding K , Koutouratsas L , Pietras C , Meyer J . Advance care planning for the homeless: a community collaboration. Narrat Inq Bioeth. 2017;7(1):E14‐E15. 10.1353/nib.2017.0028 28713126

[34] McDermott E , Selman LE . Cultural factors influencing advance care planning in progressive, incurable disease: a systematic review with narrative synthesis. J Pain Symptom Manage. 2018;56(4):613‐636. 10.1016/j.jpainsymman.2018.07.006 30025936

[35] Beddard‐Huber E , Gaspard G , Yue K . Adaptations to the serious illness conversation guide to be more culturally safe. Int J Indig Heal. 2020;16(1), 10.32799/ijih.v16i1.33192

