# Supplementary material for: Part of the solution: A survey of community organisation perspectives on barriers and facilitating actions to Advance Care Planning in British Columbia, Canada
Source: Health Expect. 2021 Dec 14;25(1):345–54. doi: 10.1111/hex.13390 (PMC8849222; doi:10.1111/hex.13390)
Supplement: Supplementary file 1 — Supporting information. [file HEX-25-345-s001.docx]

# **Appendix A: Literature Search Strategy for Barriers to Advance Care Planning**

A search was conducted for academic papers from 2015 onwards. Systematic reviews were also scanned for relevant papers.

**Search terms:**

We searched EMBASE, MEDLINE and the University of British Columbia Search Tool with the following keywords:

1. Advance Care Planning OR Advance Healthcare Planning OR Advance Medical Plan OR Advance Care (directive* or statement* or decision*) OR Living Will*

**Inclusion criteria:**

- English language
- Canadian participants
- Reporting on the patient/caregiver perspective only OR, if there are healthcare provider participants, reporting the healthcare provider perspective separately from non-healthcare provider participants

**Exclusion criteria**

- Systematic reviews, case studies, commentaries, editorials

# **Appendix B: Online Survey Questions**

## **Survey One: Advance Care Planning Priorities for BC Community Organizations- Existing Contacts**

**What is the type of your organization? (Select one)**

- Hospice Society
- Organization that supports people affected by a specific disease or illness
- Organization supporting seniors in the community
- Other (please specify) ________________________________________________

**What area of BC does your organization serve? (Select one)**

- Lower Mainland
- Fraser Valley
- Vancouver Island
- Sunshine Coast
- Interior Region
- Northern BC
- Provincial

#### **Barriers to Advance Care Planning (ACP)**

The list below contains barriers to ACP based on barriers reported by research studies.
Please select a total of six (6) of the most common barriers that you have observed in your community.
**The list may not include all barriers, so please use the text box to enter any missing common barriers. If you write in the text box, this will be counted as one of your six selections.**

- Complete lack of awareness of ACP on the part of the individual
- Confusion or lack of knowledge about how to begin or perform ACP on the part of the individual
- The belief that ACP is a one-time conversation to specify a DNR designation
- The complex terminology involved with ACP is hard to understand
- People don't speak English and don't have access to ACP resources in their own language
- People don't understand the progression or the seriousness of their own illness
- Emotional difficulty of the conversation
- Conflict with family members or hesitance of family members
- Belief that ACP is redundant because the family already knows one's wishes
- Lack of family with whom to discuss wishes
- Belief that planning for or discussing death brings bad luck or is taboo
- Mistrust of the medical system
- Health-care provider lack of time
- Health-care provider lack of knowledge
- Health-care provider lack of tact or conversation skill
- Other (please specify) ________________________________________________

Below are the six barriers that you selected in the previous question.
Drag and drop to rank your choices from most (1) to least (6) important.

*Populated based on answers to previous question.*

#### **Actions to increase Advance Care Planning (ACP) in BC**

The list below contains possible actions to increase ACP. These actions have been selected and adapted from the 2020 Pan-Canadian Advance Care Planning Framework. We hope to learn about how the items on the list apply within British Columbia. The list is separated into four categories.
Please select eight (8) actions total from among all four categories below that you think are most important to increase Advance Care Planning in BC.
The actions you select do not need to be actions that could be completed by your organization; choose actions that you think would help most to increase ACP in B.C.
The lists may not include all actions. Please enter any additional actions you feel to be important in the text box at the end (these will count towards your eight selections).

- Improve ACP literacy (e.g. walk people through ACP steps, increase training for providers)
- Develop clear, simple messages with and for target audiences
- Reframe ACP as part of life planning (e.g. build opportunities for ACP into life milestones)
- Engage communities using a wide variety of strategies (e.g. printed materials, media, influencers)
- Identify and address systemic biases as they relate to ACP (e.g racism, sexism, ablism, gender discrimination, ethnocentrism, colonization)
- Provide cultural safety and humility training to health-care providers as a way to support ACP with culturally diverse communities and populations
- Develop trusting and respectful relationships with underserved and disadvantaged communities
- Tailor tools, education and language to different underserved groups
- Define core ACP competencies and integrate them into the scope of practice, and both initial and ongoing training for all health-care providers
- Provide training to use evidence-based tools that can facilitate ACP and Goals of Care conversations
- Develop and support ACP communities of practice that build skills and strengths
- Identify champions who can be mobilized to promote ACP awareness and education (e.g. mentorship programs)
- Develop and support ACP communities of practice that build skills and strengths
- Determine how to roll up data to report on progress by organization, by jurisdiction, and at a Pan-Canadian level
- Explore the potential to build ACP questions into existing patient-reported outcomes tools
- Leverage data to make the case for ongoing investments in ACP
- Collect data on meaningful outcomes for ACP
- Invest in research and integrated knowledge translation on effective ACP programs and practices
- Require organizations and professionals to account to their accrediting organization for how ACP is delivered
- Simplify the documenting and transferring of ACP conversations
- Make the case to the federal and provincial governments for an ACP program within organizations, communities, and systems (e.g. make clear the "why" and "how" of ACP to get buy-in)
- Designate relevant departments and/or leads to champion ACP (e.g quality improvement or patient safety department, instead of off the side of someone's desk)
- Establish standards for having ACP conversations, documenting and accessing them, and translating them into medical orders
- Propose reforms to legislation to facilitate access, transferability, and recognition of ACP
- Work together with local partners to develop and adapt relevant tools and resources
- Engage diverse communities and encourage public advocacy in these communities
- Engage and include organizations who support underserved communities
- Engage partners in an integrated approach to ACP (e.g. coordinate efforts nationally, regionally, and locally)
- Integrate ACP into other relevant regional and national strategies (e.g strategies for older adults, dementia care, Indigenous health)
- Leverage partnerships strategically (e.g. home care providers may be uniquely positioned to start the ACP conversation)
- Explore potential allies in the private sector (e.g. corporate sponsors can support campaigns and resource development)
- Identify potential allies within the health system and other governmental departments (e.g engage patient safety, risk management departments, and others)
- Develop a network of key partners that already help people consider their values and think about the future (e.g. lawyers, faith-based organizations, financial planning services)
- Other (please specify) _____________________

Below are the eight actions you selected in the previous question. 
Drag and drop to rank your selected answers from most (1) to least (8) important.

*Populated based on options from previous questions.*

**Please add any comments about your ranking of the actions above.**

________________________________________________________________

________________________________________________________________

**Is delivering ACP information and facilitating ACP conversations a priority for your organization right now, given the current pandemic?**

- Yes
- No

**Please explain.**

________________________________________________________________

________________________________________________________________

**Have ACP needs in your community changed because of the COVID-19 pandemic?**

- Yes
- No

**If Yes, please describe how your community's ACP needs have changed because of the pandemic.**

________________________________________________________________

________________________________________________________________

**If Yes, what would help your organization support your community in ACP with regards to COVID-19?**

________________________________________________________________

________________________________________________________________

**Would you be willing to talk with us about your answers to this survey and your community’s ACP needs?**

We anticipate it will take up to 20 minutes, and it would be at a time convenient to you.

- Yes
- No

If Yes, Thank you! We will not use the personal information that you provide for any reason other than contacting you about your responses to this survey.

Name ________________________________________________

Phone number ________________________________________________

Email _______________________________________________

Survey Two: **Advance Care Planning Priorities for BC Community Organizations- New and Non-Active Contacts**

**1. Is your organization a non-profit in BC? (Y/N)**

*[If no:]*

Thank you for taking the time to complete our survey. At this time, we are only looking to hear from British Columbian community **non-profits** engaging or wanting to engage their communities in Advance Care Planning. However, we anticipate expanding this to other types of organisations in the future. If you have any questions or concerns, please reach out to Ellie Siden, Advance Care Planning Student, at [esiden@bc-cpc.ca](mailto:esiden@bc-cpc.ca).

*[go to Q19]*

*[If yes, continue to Q2]*

**2. Before receiving the invitation for this survey, had you or your organization heard of Advance Care Planning?**

**What is Advance Care Planning?**

Advance Care Planning is a process of thinking and talking about your future health-care decisions with the people you trust. The goal is for you to get the care that’s right for you, even if you’re unable to speak for yourself.

For more information on Advance Care Planning, visit our website.

- Yes
- No

*[If no:]*

Thank you for taking the time to complete our survey. At this time, we are only looking to hear from British Columbian community non-profits **familiar with Advance Care Planning**. However, we anticipate expanding this to other types of organisations in the future. If you have any questions or concerns, please reach out to Ellie Siden, Advance Care Planning Student, at [esiden@bc-cpc.ca](mailto:esiden@bc-cpc.ca).

*[go to Q19]*

*[If yes, continue to Q3]*

**3. Does your organization support Advance Care Planning in your community? (Y/N)**

Examples of supporting Advance Care Planning:

- Providing Advance Care Planning resources in the form of brochures, web pages, pdfs, or other
- Offering Advance Care Planning workshops, trainings, or one-on-one conversations
- Referring clients or community members to the ACP resources of other organizations

*[If yes, continue to Q4]*

*[If no, continue to Q5]*

**4. In what ways does your organization support Advance Care Planning? (Select all that apply)**

- Hard-copy or online resources developed by my organization
- Hard-copy or online resources developed by another organization
- ACP-related referrals to legal or medical professionals
- ACP education for the public
- ACP education for health-care providers
- Other (please specify)

**5. What is the type of your organization? (Select one)**

- Hospice Society
- Organization that supports people affected by a specific disease or illness
- Organization supporting seniors in the community
- Organization focusing on the law and legal access
- Faith-based organization (please specify)
- Other (please specify) ________________________________________________

**6. Which area of BC does your organization serve? (Select one)**

- Lower Mainland
- Fraser Valley
- Vancouver Island
- Sunshine Coast
- Interior Region
- Northern BC
- Provincial

#### **Barriers to Advance Care Planning (ACP)**

7. The list below contains barriers to Advance Care Planning based on barriers reported by research studies.
Please select a total of six (6) of the most common barriers that you have observed in your community.
The list may not include all barriers, so please use the text box to enter any missing common barriers. If you write in the text box, this will be counted as one of your six selections.

- Complete lack of awareness of ACP on the part of the individual
- Confusion or lack of knowledge about how to begin or perform ACP on the part of the individual
- The belief that ACP is a one-time conversation to specify a DNR designation
- The complex terminology involved with ACP is hard to understand
- People don't speak English and don't have access to ACP resources in their own language
- People don't understand the progression or the seriousness of their own illness
- Emotional difficulty of the conversation
- Conflict with family members or hesitance of family members
- Belief that ACP is redundant because the family already knows one's wishes
- Lack of family with whom to discuss wishes
- Belief that planning for or discussing death brings bad luck or is taboo
- Mistrust of the medical system
- Health-care provider lack of time
- Health-care provider lack of knowledge
- Health-care provider lack of tact or conversation skill
- Other (please specify) ________________________________________________

8. Below are the six barriers that you selected in the previous question.
Drag and drop to rank your choices from most (1) to least (6) important.

*Populated based on answers to previous question.*

#### **Actions to increase Advance Care Planning (ACP) in BC**

9. The list below contains possible actions to increase ACP. These actions have been selected and adapted from the [2020 Pan-Canadian Advance Care Planning Framework](https://www.advancecareplanning.ca/acp-framework/). We hope to learn about how the items on the list apply within British Columbia. The list is separated into four categories.
Please select eight (8) actions total from among all four categories below that you think are most important to increase Advance Care Planning in BC.
The actions you select do not need to be actions that could be completed by your organization; choose actions that you think would help most to increase ACP in B.C.
The lists may not include all actions. Please enter any additional actions you feel to be important in the text box at the end (these will count towards your eight selections).

- Improve ACP literacy (e.g. walk people through ACP steps, increase training for providers)
- Develop clear, simple messages with and for target audiences
- Reframe ACP as part of life planning (e.g. build opportunities for ACP into life milestones)
- Engage communities using a wide variety of strategies (e.g. printed materials, media, influencers)
- Identify and address systemic biases as they relate to ACP (e.g racism, sexism, ableism, gender discrimination, ethnocentrism, colonization)
- Provide cultural safety and humility training to health-care providers as a way to support ACP with culturally diverse communities and populations
- Develop trusting and respectful relationships with underserved and disadvantaged communities (7)
- Tailor tools, education and language to different underserved groups
- Define core ACP competencies and integrate them into the scope of practice, and both initial and ongoing training for all health-care providers
- Provide training to use evidence-based tools that can facilitate ACP and Goals of Care conversations
- Develop and support ACP communities of practice that build skills and strengths
- Identify champions who can be mobilized to promote ACP awareness and education (e.g. mentorship programs)
- Develop and support ACP communities of practice that build skills and strengths
- Determine how to roll up data to report on progress by organization, by jurisdiction, and at a Pan-Canadian level
- Explore the potential to build ACP questions into existing patient-reported outcomes tools
- Leverage data to make the case for ongoing investments in ACP
- Collect data on meaningful outcomes for ACP
- Invest in research and integrated knowledge translation on effective ACP programs and practices
- Require organizations and professionals to account to their accrediting organization for how ACP is delivered
- Simplify the documenting and transferring of ACP conversations
- Make the case to the federal and provincial governments for an ACP program within organizations, communities, and systems (e.g. make clear the "why" and "how" of ACP to get buy-in)
- Designate relevant departments and/or leads to champion ACP (e.g quality improvement or patient safety department, instead of off the side of someone's desk)
- Establish standards for having ACP conversations, documenting and accessing them, and translating them into medical orders
- Propose reforms to legislation to facilitate access, transferability, and recognition of ACP
- Work together with local partners to develop and adapt relevant tools and resources
- Engage diverse communities and encourage public advocacy in these communities
- Engage and include organizations who support underserved communities
- Engage partners in an integrated approach to ACP (e.g. coordinate efforts nationally, regionally, and locally)
- Integrate ACP into other relevant regional and national strategies (e.g strategies for older adults, dementia care, Indigenous health)
- Leverage partnerships strategically (e.g. home care providers may be uniquely positioned to start the ACP conversation)
- Explore potential allies in the private sector (e.g. corporate sponsors can support campaigns and resource development)
- Identify potential allies within the health system and other governmental departments (e.g engage patient safety, risk management departments, and others)
- Develop a network of key partners that already help people consider their values and think about the future (e.g. lawyers, faith-based organizations, financial planning services)
- Other (please specify) _____________________

10. Below are the eight actions you selected in the previous question.
Drag and drop to rank your selected answers from most (1) to least (8) important.

*Populated based on options from previous questions.*

**11. Please add any comments about your ranking of the actions above.**

________________________________________________________________

________________________________________________________________

**12.** *[only if answered yes to Q3]* **Is delivering Advance Care Planning information and facilitating Advance Care Planning conversations a priority for your organization right now, given the current pandemic?**

- Yes
- No

**13.** *[only if answered yes to Q3]* **Please explain.**

________________________________________________________________

________________________________________________________________

**14.** *[only if answered yes to Q3]* **Have Advance Care Planning needs in your community changed because of the COVID-19 pandemic?**

- Yes
- No

**15.** *[only if answered yes to Q3 and Q14]* **If Yes, please describe how your community's Advance Care Planning needs have changed because of the pandemic.**

________________________________________________________________

________________________________________________________________

**16.** *[only if answered yes to Q3 and Q14]* **If Yes, what would help your organization support your community in Advance Care Planning with regards to COVID-19?**

________________________________________________________________

________________________________________________________________

**17. Would you be willing to talk with us about your answers to this survey and your community’s Advance Care Planning needs?**

We anticipate it will take up to 20 minutes, and it would be at a time convenient to you.

- Yes
- No

*[If yes, continue to Q18]*

*[If no go to Q19]*

**18. Thank you! We will not use the personal information that you provide for any reason other than contacting you about your responses to this survey, unless you indicate below that you would like to remain in contact with us.**

Your personal information will not be linked to your survey responses.

Name ________________________________________________

Phone number ________________________________________________

Email _______________________________________________

Organization _________________________________________

**Stay connected through our mailing list**

By signing up for our BCCPC community mailing list, you will be notified about grants and other Advance Care Planning resources that may help you to support your community.

- Yes! I would like to be added to the BC Centre for Palliative Care’s community mailing list. *Please ensure that you have entered your contact information above.*

We appreciate your help! Thank you so much for completing our survey. A summary report of our findings will be made public on our website when complete. If you have any questions or comments about this project, please feel free to reach out at any time to Ellie Siden at [esiden@bc-cpc.ca](mailto:esiden@bc-cpc.ca).

**[SURVEY END]**

**19. Stay connected through our mailing list**

By signing up for our BCCPC community mailing list, you will be notified about grants and other Advance Care Planning resources that may help you to support your community.

- Yes! I would like to be added to the BC Centre for Palliative Care’s community mailing list

If so, please provide your name and contact information to be added to our mailing list. Your personal information will not be linked to your survey responses.

- Name (<First> <Last>)
- Email
- Organization

[shows if Q1 answered No] **What is Advance Care Planning?**

Advance Care Planning is a process of thinking and talking about your future health-care decisions with the people you trust. The goal is for you to get the care that’s right for you, even if you’re unable to speak for yourself.

For more information on Advance Care Planning, visit our website.

**[SURVEY END]**

**Appendix C: Interview Script**

## **1) Barriers**

- Looking at the first part of the survey, can you explain what you feel to be the biggest barrier to ACP in your community and why?
  - Has this always been the case/ is this new?
  - What experiences lead you to feel this to be the case?  
    (e.g. talking with people/own interactions with ACP/ literature)
- Are there any important barriers that you feel to be missing?
- Do you feel like any of these are being addressed currently or have been addressed with initiatives in the past?

## **2) Facilitators**

- Looking at the second part of the survey, your ranking of actions, can you walk me through your ranking process?
  - Tell me about the actions that you ranked first and second.
- Do you think that these actions are important to all communities, or is there something about yours that makes these actions particularly relevant?
- Do you feel your top two actions to be feasible? Why or why not?
- Do you have an idea how your top two choices might be brought about? Who would need to be involved for them to happen?
- Were there any important actions that you felt needed to be added to the list? If so, can you tell me what they are and why?
- Do you feel like any of these are being addressed currently or have been addressed with initiatives in the past?

## **3) COVID-19**

- Do you have anything that you would like to add to your written response about how COVID may have affected your community’s ACP needs?

## **4) Closing**

- Is there anything more that you would like to tell me about Advance Care Planning in your community?
- Do you have any questions for me?

# **Appendix D: Quantitative Facilitators to Advance Care Planning**

**Table 3: Weighted ranking of most important actions to increase advance care planning in British Columbia from a community-based non-profit perspective**

| Facilitator | Weighted Score | Number of participants ranking facilitator (n=48)  n (%) |
| --- | --- | --- |
| Develop clear, simple messages with and for target audiences | 151 | 21 (44) |
| Improve ACP literacy (e.g. walk people through ACP steps, increase training for providers) | 150 | 23 (48) |
| Reframe ACP as part of life planning (e.g. build opportunities for ACP into life milestones) | 140 | 22 (45) |
| Simplify the documenting and transferring of ACP conversations | 108 | 21 (44) |
| Define core ACP competencies and integrate them into the scope of practice, and both initial and ongoing training for all health-care providers | 88 | 17 (35) |
| Work together with local partners to develop and adapt relevant tools and resources | 84 | 19 (40) |
| Establish standards for having ACP conversations, documenting and accessing them, and translating them into medical orders | 81 | 20 (42) |
| Develop a network of key partners that already help people consider their values and think about the future (e.g. lawyers, faith-based organizations, financial planning services) | 70.5 | 20 (42) |
| Provide cultural safety and humility training to health-care providers as a way to support ACP with culturally diverse communities and populations | 68.5 | 15 (31) |
| Identify champions who can be mobilized to promote ACP awareness and education (e.g. mentorship programs) | 66 | 15 (31) |
| Integrate ACP into other relevant regional and national strategies (e.g strategies for older adults, dementia care, Indigenous health) | 62 | 21 (44) |
| Develop trusting and respectful relationships with underserved and disadvantaged communities | 58 | 12 (25) |
| Identify and address systemic biases as they relate to ACP (e.g racism, sexism, ablism, gender discrimination, ethnocentrism, colonization) | 53 | 10 (21) |
| Identify potential allies within the health system and other governmental departments (e.g engage patient safety, risk management departments, and others) | 44.5 | 11 (23) |
| Explore the potential to build ACP questions into existing patient-reported outcomes tools | 42.5 | 12 (25) |
| Tailor tools, education and language to different underserved groups | 42 | 12 (25) |
| Provide training to use evidence-based tools that can facilitate ACP and Goals of Care conversations | 41.5 | 10 (21) |
| Develop and support ACP communities of practice that build skills and strengths | 37 | 15 (31) |
| Engage and include organizations who support underserved communities | 34.5 | 9 (19) |
| Leverage partnerships strategically (e.g. home care providers may be uniquely positioned to start the ACP conversation) | 33 | 11 (23) |
| Engage communities using a wide variety of strategies (e.g. printed materials, media, influencers) | 30.5 | 6 (13) |
| Engage partners in an integrated approach to ACP (e.g. coordinate efforts nationally, regionally, and locally) | 30 | 7 (15) |
| Collect data on meaningful outcomes for ACP | 29.5 | 11 (23) |
| Make the case to the federal and provincial governments for an ACP program within organizations, communities, and systems (e.g. make clear the "why" and "how" of ACP to get buy-in) | 26 | 7 (15) |
| Engage diverse communities and encourage public advocacy in these communities | 20.5 | 10 (21) |
| Invest in research and integrated knowledge translation on effective ACP programs and practices | 20 | 6 (13) |
| Designate relevant departments and/or leads to champion ACP (e.g. quality improvement or patient safety department, instead of off the side of someone's desk) | 20 | 4 (8) |
| Require organizations and professionals to account to their accrediting organization for how ACP is delivered | 19 | 5 (10) |
| Leverage data to make the case for ongoing investments in ACP | 13 | 5 (10) |
| Explore potential allies in the private sector (e.g. corporate sponsors can support campaigns and resource development) | 10.5 | 2 (4) |
| Other (please specify) | 9 | 2 (4) |
| Propose reforms to legislation to facilitate access, transferability, and recognition of ACP | 7 | 2 (4) |
| Determine how to roll up data to report on progress by organization, by jurisdiction, and at a Pan-Canadian level | 6 | 1 (2) |

**Abbreviations: ACP = Advance care planning; DNR = Do-Not-Resuscitate**
